# Integration of Smart Home and Building Automation Systems in Virtual Reality and Robotics-Based Technological Environment for Neurorehabilitation: A Pilot Study Protocol

**DOI:** 10.3390/jpm14050522

**Published:** 2024-05-14

**Authors:** Sara Federico, Mirko Zitti, Martina Regazzetti, Enrico Dal Pozzo, Błażej Cieślik, Alberto Pomella, Francesca Stival, Marco Pirini, Giorgia Pregnolato, Pawel Kiper

**Affiliations:** 1 Healthcare Innovation Technology Lab, IRCCS San Camillo Hospital, 30126 Venice, Italy; sara.federico@hsancamillo.it (S.F.); mirko.zitti@hsancamillo.it (M.Z.); martina.regazzetti@hsancamillo.it (M.R.); enrico.dalpozzo@hsancamillo.it (E.D.P.); giorgia.pregnolato@hsancamillo.it (G.P.); 2 VIMAR SpA, 36063 Marostica, Italy; alberto.pomella@vimar.com (A.P.); francesca.stival@vimar.com (F.S.); 3 Khymeia Group, 35129 Padua, Italy; m.pirini@khymeia.com

**Keywords:** digital health, virtual reality, robotics, home automation, building automation, smart home technology, neurological rehabilitation

## Abstract

Technological innovation has revolutionized healthcare, particularly in neurological rehabilitation, where it has been used to address chronic conditions. Smart home and building automation (SH&BA) technologies offer promising solutions for managing chronic disabilities associated with such conditions. This single group, pre-post longitudinal pilot study, part of the H2020 HosmartAI project, aims to explore the integration of smart home technologies into neurorehabilitation. Eighty subjects will be enrolled from IRCCS San Camillo Hospital (Venice, Italy) and will receive rehabilitation treatment through virtual reality (VR) and robotics devices for 15 h per day, 5 days a week for 3 weeks in the HosmartAI Room (HR), equipped with SH&BA devices measuring the environment. The study seeks to optimize patient outcomes and refine rehabilitation practices. Findings will be disseminated through peer-reviewed publications and scientific meetings, contributing to advancements in neurological rehabilitation and guiding future research.

## 1. Introduction

Technological innovation has significantly reshaped various aspects of daily life, including healthcare, where advancements are continually improving patient care and outcomes. One notable area of progress is the integration of technology to enhance rehabilitation for individuals with neurological disorders [1,2]. This integration involves leveraging robotics, virtual reality devices, and sensor technologies to augment traditional rehabilitation methods and facilitate motor-function recovery [3]. These technologies not only promote patient engagement but also offer real-time monitoring capabilities, allowing for more personalized and effective treatment approaches [4].

Neurologic conditions often result in long-term disabilities, necessitating innovative solutions to enhance patient well-being and safety. Among these solutions, smart home and building automation systems (SH&BA) technologies have emerged as promising tools [5]. By monitoring patients’ health in real-time and providing data to healthcare professionals, smart homes enable proactive disease management, reducing the risk of exacerbations [6]. Moreover, these technologies promote patient independence and safety, aligning with the core objective of managing chronic conditions effectively [7]. Integrating smart homes within communities further enhances care continuity and enables timely interventions for older adults [8]. Recent technological advancements have opened new avenues for enhancing the effectiveness and accessibility of rehabilitation therapies for individuals with neurological disabilities. Among these innovations, the integration of SH&BA systems into neurorehabilitation therapies emerges as a promising opportunity to optimize rehabilitation outcomes. SH&BA systems, through the capability of monitoring, automation, and control of household devices and environments, provides an ideal context to extend the scope of rehabilitation therapies beyond traditional clinical settings. This innovative approach enables the creation of a safe, controlled, and personalized living environment that actively supports individuals through their rehabilitation journey, facilitating continuous practice and remote progress monitoring [6,7]. In this context, the integration of smart-home devices into rehabilitation therapies presents itself as a key strategy to improve effectiveness, efficiency, and overall patient experience during the neurological recovery process. This pilot study protocol aims to explore in detail the benefits and challenges of such integration, providing an empirical basis for its development and implementation in both clinical and home settings. Despite the promising results, to our best knowledge, the full potential of SH&BA systems in healthcare remains underexplored, with ongoing research efforts focused on uncovering new applications and optimizing existing systems.

Hence, the present single group-longitudinal pilot study, conducted within the H2020 “Hospital Smart development based on AI” (HosmartAI) project, aims to explore the impact of integrating smart-home sensors and actuators in a neurorehabilitation hospital by motor-recovery treatment uptake. It also seeks to assess the clinical efficacy and cost-effectiveness of motor treatment within a dedicated rehabilitation room (HosmartAI Room, HR) equipped with smart-home devices. Additionally, the study aims to investigate the usability and acceptability of such sensor integration among users and healthcare providers, with a view to improving the overall rehabilitation experience and optimizing patient outcomes. The possibility of applying motion detection and environmental control sensors in technological rehabilitation rooms could allow for the synchronization of environmental data with a patient’s clinical history to study functional recovery.

## 2. Materials and Methods

### 2.1. Study Setting and Design

To ensure the completeness and quality of reporting, we referred to the SPIRIT (Standard Protocol Items: Recommendations for Interventional Trials) [9] guidelines as well as Thabane and Lancaster’s [10] guidance on how to report protocols of pilot and feasibility trials. The protocol was registered in the ClinicalTrials.gov PRS database (Registration number: NCT06270420, https://clinicaltrials.gov/study/NCT06270420, accessed on 22 February 2024). This single group, pre-post longitudinal pilot study will be conducted within the HosmartAI research project (EU Horizon 2020, research and innovation program—grant agreement No 101016834). The trial will last twenty months and will be carried out at the San Camillo IRCCS Hospital (Venice, Italy), within which a smart rehabilitative room (HR) will be created. This room will feature rehabilitation devices complemented by smart-home sensors and actuators developed by VIMAR SpA (Marostica, Italy), all of which send data to a cloud system (Kloud) created by Khymeia Group (Padua, Italy). The purpose of this setup is to develop an innovative solution to monitor various activities that patients can perform autonomously or under therapists’ supervision. This solution aims to assist clinicians in monitoring patients within the hospital, identifying potential risks, and ensuring adherence to therapy protocols. Throughout the pilot phase, stakeholders involved will be closely observed using a persona-based approach, providing valuable insights into the behaviors of therapists and patients, thus enabling the analysis and exploration of the extension of active patient monitoring to the home environment.

Within the HR, various rehabilitative treatments will be conducted using different innovative devices. These will include the AMADEO^®^ system (Tyromotion GmbH, Graz, Austria), which is an upper limb rehabilitation robot enabling selective hand and finger movements. Additionally, the PABLO^®^ virtual reality rehabilitation system (Tyromotion GmbH), will provide immersive tasks with real-time biofeedback. The VRRS EVO^®^ (Virtual Reality Rehabilitation System, Khymeia Group Ltd., Padua, Italy) will offer virtual scenarios for kinematic tasks to be emulated by patient’s real arm movements while controlling a virtual object via a motion tracking system. Lastly, the OAK^®^ integrated virtual reality system (Khymeia Group Ltd.) will be used for fall risk assessment and prevention, enhancing balance and stability.

Additionally, the room will be equipped with smart-home sensors and actuators designed to gather environmental data and information, which can be conveniently accessed and managed by the physiotherapist through a digital interface. Developed by VIMAR SpA, these devices will encompass functions such as room occupancy monitoring (e.g., utilizing access control badges), tracking therapy dosage, detecting motion, monitoring temperature and light levels, and overseeing shutter position, as well as detecting falls. These smart-home devices aim to collect comprehensive data regarding various aspects of the environment within the healthcare facility. Physiotherapists will utilize a smartphone app (i.e., Vimar VIEW App, version 2.8.2 for Android and version 2.8.1 for iOS) to remotely oversee the HR’s environment and monitor patients’ activities. Moreover, the cloud system (i.e., Kloud, Khymeia Group Ltd.) will seamlessly integrate data coming from these devices, facilitating efficient data management and analysis to optimize patient care and rehabilitation outcomes.

### 2.2. Participants and Eligibility Criteria

The study will enroll 80 subjects among the patients hospitalized at the IRCCS San Camillo Hospital in Venice. Inclusion criteria for enrolment include an age greater than 18 years and a diagnosis of neurological pathology, such as ischemic and/or hemorrhagic stroke, Parkinson’s disease, multiple sclerosis, head trauma, brain tumor, ataxia, post-COVID syndrome, or peripheral neuropathy. Exclusion criteria include non-stabilized fractures, severe depression, significant visual and/or hearing impairments, dementia, uncontrolled epilepsy, severe neglect, and profound comprehension deficits that would hinder interactions with robotic and virtual reality devices.

### 2.3. Intervention

**Figure 1:**
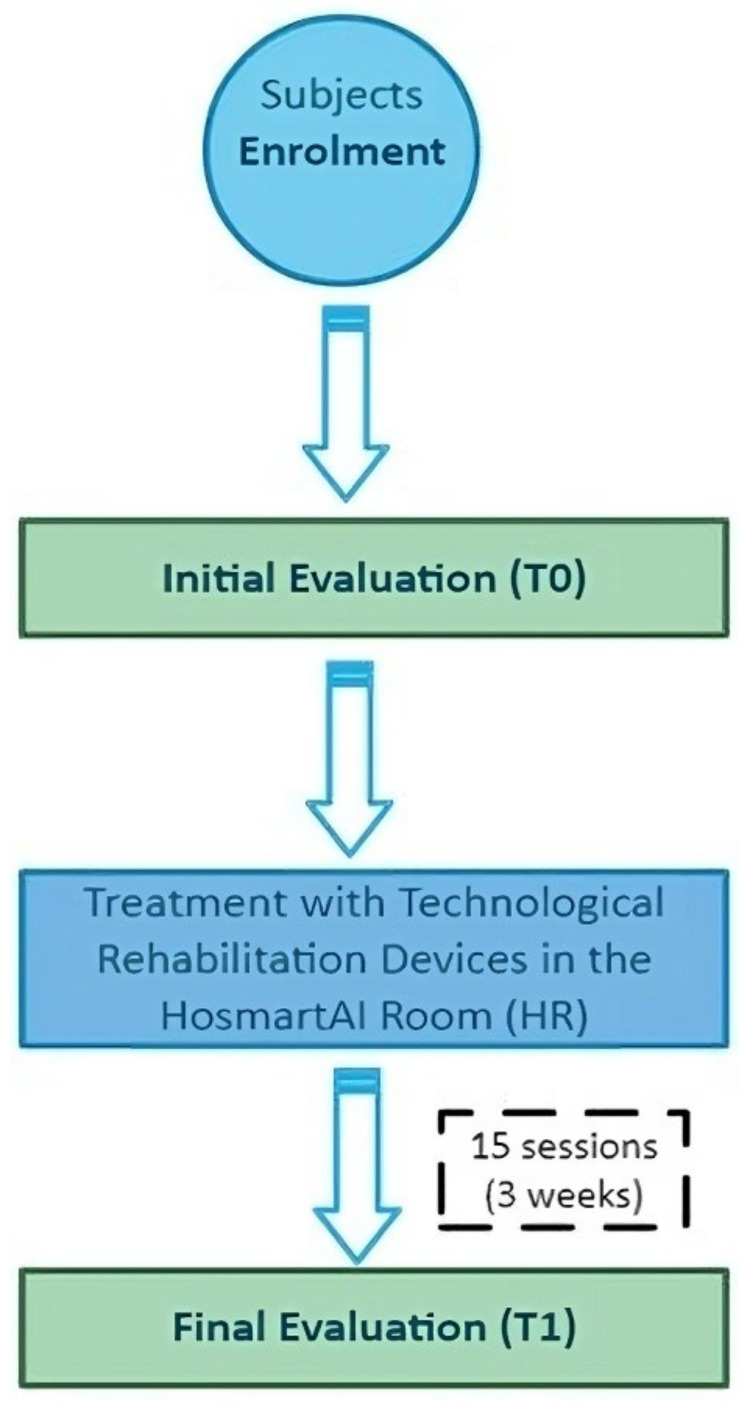
Participants Timeline.

Subjects were enrolled among hospitalized patients based on the pre-defined inclusion criteria. The identification of eligible subjects was conducted through notification by the Clinical Research Unit at San Camillo Hospital. Subsequently, researchers from the HosmartAI project will enroll and evaluate (T0) the patients, after which they will commence their experimental treatment in the HR room, in addition to their regular daily conventional rehabilitation session. The experimental treatment will be tailored to the subjects’ therapeutic needs and will run for three weeks, comprising 15 one-hour sessions each, conducted five days a week. The specialized devices targeting balance, manual dexterity, and limb functionality, as described in the study setting section, will be utilized during these sessions. Pre-treatment (T0) and post-treatment (T1) assessments will be conducted using validated clinical scales and tests to determine therapy effects. Additionally, device-specific evaluation systems will be employed to assess treatment outcomes. A qualitative analysis will ascertain technology usability, acceptability, and user satisfaction. All assessments and treatments will be administered by qualified physiotherapists. Figure 1 illustrates the participants’ timeline.

### 2.4. Outcome and Timelines

The primary aim of the study is to verify the productivity of the rehabilitation service at the IRCCS San Camillo Hospital following the sensorization of the HR with the smart-home system’s devices. Productivity outcomes will be measured through treatment performance, physiotherapists involvement, and adverse events. Assessments will be conducted monthly over a period of one year; the specifics are listed below.

Treatment Performance:

- Direct measurements of treatments provided in the HR, such as number of treatments provided per month; number of times accessed per month by system users (i.e., patients); number of therapy minutes provided per month and overall therapy duration.

Physiotherapist Involvement:

- The number of physiotherapists providing treatment relative to the number of patients will be quantified. This ratio (number of physiotherapists/number of patients), will reflect the allocation of staff resources during the study period.

Adverse Events:

- Measurement and recording of adverse events will involve number of falls occurring during the observation period within the HR.

The following secondary outcomes, changes in clinical parameters following therapies conducted in the HR, as well as satisfaction and user experience associated with the therapy, will also be assessed at baseline (T0) and at the end of the treatment, after 3 weeks (T1).

Changes in motor function:

- For change in upper limb function: The Box and Blocks Test [11] is a measurement of manual dexterity and gross motor skills. Participants move blocks from one side of a divided box to the other within one minute. The number of blocks transferred successfully serves as a measure of manual dexterity and motor function, with higher scores indicating better performance. The Nine Hole Pegboard Test (NHPT) [12] is a quantitative evaluation employed to assess finger dexterity and motor coordination. During the NHPT the participant is required to pick up nine pegs one at a time from a container, place them into nine holes on a pegboard, and then remove them again as quickly as possible. The time taken to complete the task is recorded, with a shorter completion time indicating better manual dexterity and hand function. The Reaching Performance Scale (RPS) [13] is a clinical assessment tool used to evaluate upper limb function, specifically reaching ability. The RPS consists of a set of six standardized tasks related to reaching for and grasping objects. The therapist is asked to decompose the reaching movement into its elements reaching a close (18 points) and far target (18 points), with each of the six movement components being scored from 0 to 3 and with a higher value indicating better performance.
- For lower limb function and walking: The Ten Meters Walk Test (10 mWT) [14] is used to assess walking speed in meters/second (m/s) over a short distance. Functional Ambulation Categories [5,15] is a 6-point assessment tool used to evaluate walking ability. FAC assesses the amount of human support a patient requires during walking, irrespective of their use of assistive devices. Scores range from 0 to 5, with higher scores indicating greater independence and lower levels of assistance required during walking.
- For trunk control and balance assessment: The Trunk Control Test [16] is a clinical assessment tool used to evaluate trunk control consisting of four items, each scored from 0 (unable to perform) to 25 (normal performance), with intermediate scores for partial performance. The total score is the sum of scores from all four tasks. The Berg Balance Scale (BBS) [17,18] is a 14-item objective measure designed to assess static balance and fall risk in adults. Each item is scored on a scale from 0 to 4, with a maximum total score of 56. Higher scores indicate better balance abilities.

Change in Health-related quality of Life (HRQoL) levels:

- The three-level version of EQ-5D (EQ-5D-3L) [19] is a standardized instrument for measuring health-related quality of life that consists of a descriptive system and a visual analogue scale (VAS). The descriptive system comprises five dimensions of health: mobility, self-care, usual activities, pain/discomfort, and anxiety/depression. Each dimension has three levels of severity: no problems, some problems, and extreme problems. The VAS records the respondent’s self-rated health on a scale from 0 (worst imaginable health state) to 100 (best imaginable health state).

Assessment of user experience and satisfaction:

- User satisfaction with the treatments provided in the HR will be assessed using the Short Form Patient Satisfaction Questionnaire (PSQ-18) [20], a self-reported 18-item questionnaire with a Likert scale ranging from 1 (strongly agree) to 6 (strongly disagree). It assesses patient satisfaction with healthcare services, investigating quality of care received, communication with healthcare providers, accessibility of services, and overall satisfaction with the healthcare experience.
- The System Usability Scale (SUS) [21] provides a quantitative measure of perceived user satisfaction and usability of a system, product, or a service. It consists of 10 items, each rated on a 5-point Likert scale ranging from “strongly agree” to “strongly disagree”. Scores range from 0 to 100, with higher scores indicating greater perceived usability. The User Experience Questionnaire (UEQ) [22] is an end-user questionnaire that measures a comprehensive impression of the product user experience, investigating the following dimensions: attractiveness, perspicuity, efficiency, dependability, stimulation, and novelty. It consists of 26 Likert-scale questions ranging from 1 to 7 points.

### 2.5. Sample Size

The study aims to recruit subjects from IRCCS San Camillo Hospital’s Neurorehabilitation Department. With an average occupancy of 60 beds per week, it is estimated that four eligible subjects are admitted monthly. Given a 20-month trial period, the target sample size was set at 80 subjects. To evaluate the reliability of our study’s results, a retrospective (post-hoc) power analysis was conducted using G*Power 3.1.9.6 software (Heinrich-Heine-University Düsseldorf, Germany). With a two-tailed test, a moderate effect size (Cohen’s d) of 0.5, an alpha level of 0.05, and a total sample size of 80, the analysis yielded a calculated statistical power of 0.99. This suggests a strong probability of detecting the expected effects and highlights the potential dependability of our findings.

### 2.6. Data Collection and Analysis Plan

Patient data, including clinical and demographic information, will be collected and inputted into treatment devices. A unified cloud platform will consolidate information from SH&BA devices and patient clinical data stored across devices. Data from HR sensors will gauge patient therapy adherence.

Descriptive analysis will be initially conducted on the enrolled subjects’ sample. For each demographic and clinical variable, we will consider the mean, standard deviation [23], absolute frequencies (n), and percentages (%). For each mode of clinical treatment delivery (i.e., depending on the physiotherapist/patient ratio), pre-treatment (T0) and post-treatment (T1) motor performance values will be compared using either Student’s t-test for paired data or the Wilcoxon test, depending on the data distribution, which will be assessed with the Kolmogorov–Smirnov test. To assess the presence of a statistically significant difference in motor recovery in relation to the type of treatment model, the motor outcomes of the patients will be compared by means of parametric or non-parametric independent sample comparison tests (i.e., Student’s t-test for independent data or the Mann–Whitney test), depending on the data distribution. To identify the patient profile that best fits each therapy model, regression models will be utilized, with the type of therapy model as the dependent variable, and assessment scales and anamnestic variables as predictor variables. To define the satisfaction, usability and technology acceptance and clinical profile of patients accessing technological therapy, tests for customer satisfaction will be utilized. The statistical significance is set at *p* < 0.05. Data analysis will be conducted using the free statistical software R Studio, version 4.0.3 [24].

## 3. Discussion

This pilot study protocol outlines a structured approach to investigate the integration of SH&BA systems in neurorehabilitation, aiming to provide insights into the potential benefits and challenges of this innovative approach. Findings from this pilot study may inform the design of larger-scale trials and guide the implementation of smart-home technology in clinical practice, ultimately enhancing rehabilitation outcomes for individuals with neurological impairments.

With the ongoing advancements in digital healthcare, integrating into rehabilitation processes is crucial for enhancing patient care outcomes and management efficiency [6,7,8]. This study aims to combine technology with clinical requirements by sensorizing the environment. This enables the tracking of the number of eligible patients treated, the actual physiotherapy hours, and the ratio of physiotherapists to patients, all within existing financial constraints. By comparing pre-project data with the acquired results, potential savings of around 20–30% for the facility can be estimated. However, realizing the full potential of smart-home technologies in healthcare requires robust research methodologies and ethical considerations regarding data privacy. Continued research and development in these areas are essential to further optimize these technologies and maximize their benefits for patients and healthcare systems alike [25,26,27]. The study’s novelty lies in achieving cost savings while increasing productivity without compromising clinical outcomes. Personalized treatment remains paramount, ensuring patient-centric care through tailored device selection, exercise settings, and session management. Thus, even in the worst-case scenario, clinical outcomes are expected to be maintained, if not improved. Additionally, assessing device usability and patient satisfaction will validate the model’s viability. This project addresses the pressing need for increased therapy hours without escalating healthcare costs. Recruiting staff can be challenging, particularly in hard-to-reach areas, yet high-intensity rehabilitation centers, like San Camillo Hospital, must prioritize therapy hours. Balancing these needs requires economically feasible and qualitatively robust solutions, which this trial aims to provide.

Future implications include profiling patient suitability for this organizational model and its transferability to other rehabilitation settings. However, it is important to acknowledge the limitations of this study, such as the need for expert physiotherapists and potential enrolment complexities, as well as the inherent constraints of pilot studies, including small sample size, a lack of control groups, and limited generalizability. Nevertheless, despite these limitations, the study’s findings are expected to inform and facilitate similar technology implementations in various rehabilitation medical centers. One of the most significant implications is the potential for personalized therapeutic approaches which could concurrently enhance operational efficiency. Specifically, the implementation of SH&BA therapies within technological group treatments for neurological subjects holds promise for augmenting therapy quantity, thereby potentially improving clinical outcomes [28]. This envisaged scenario would unfold alongside resource optimization efforts, aiming to enhance the precision and reliability of collected data and administered therapies, while also striving to mitigate errors. Furthermore, enhancing the patient experience is indeed a significant aspect. The integration of smart-home technologies provides the potential to enhance the overall patient experience during the rehabilitation process. By offering a more comfortable and familiar environment for therapy, it enables greater autonomy and control throughout the healing journey. Moreover, the consideration of patients’ social interaction during group therapy sessions, which also contributes to clinical improvement, further underscores the holistic approach to patient care [29]. Lastly, the remote monitoring capabilities of these systems could expand access to rehabilitation services, particularly for patients in remote or disadvantaged areas.

Disseminating these results nationally and internationally underscores the project’s significance. Efforts to address these limitations and refine the methodology will be crucial in maximizing the impact and applicability of the study’s findings.

## 4. Ethics and Dissemination

This study was approved by the Ethics Committee for Clinical Trials (CESC) of the Province of Venice and IRCSS San Camillo Ethics Committee (ID: 1469/IRCCS San Camillo). The study findings will be published in international peer-reviewed scientific journals and presented at local and international scientific meetings. Summarized results will also be provided to healthcare professionals and other relevant stakeholders as well as the general public.

## Data Availability

Data will be available upon request to the corresponding author.
